# Testing if vitamin K1 reduces the progression of non-severe calcific aortic stenosis: design and rationale of the Prevention of Aortic Stenosis Progression Phylloquinone Ossification Reduction Trial (PASSPORT)

**DOI:** 10.1093/ehjopen/oeaf144

**Published:** 2025-12-05

**Authors:** William A Courtney, Jarryd Walker, Aindreas Dorai-Raj, Tom Gilbert, Adil Rajwani, Jamie W Bellinge, Jonathan M Hodgson, Graham S Hillis, Carl J Schultz

**Affiliations:** Medical School, University of Western Australia, 35 Stirling Highway, Crawley, Perth, WA 6009, Australia; Department of Cardiology, Royal Perth Hospital, Victoria Square, Perth, WA 6000, Australia; Department of Cardiology, Royal Perth Hospital, Victoria Square, Perth, WA 6000, Australia; Department of Cardiology, Royal Perth Hospital, Victoria Square, Perth, WA 6000, Australia; Department of Cardiology, Royal Perth Hospital, Victoria Square, Perth, WA 6000, Australia; Department of Cardiology, Royal Perth Hospital, Victoria Square, Perth, WA 6000, Australia; Medical School, Curtin University, Bentley, Perth, WA 6102, Australia; Medical School, University of Western Australia, 35 Stirling Highway, Crawley, Perth, WA 6009, Australia; Department of Nuclear Medicine, Sir Charles Gairdner Hospital, Nedlands, Perth, WA 6009, Australia; Medical School, University of Western Australia, 35 Stirling Highway, Crawley, Perth, WA 6009, Australia; School of Medical and Health Sciences, Edith Cowan University, Joondalup, WA 6027, Australia; Medical School, University of Western Australia, 35 Stirling Highway, Crawley, Perth, WA 6009, Australia; Department of Cardiology, Royal Perth Hospital, Victoria Square, Perth, WA 6000, Australia; Medical School, University of Western Australia, 35 Stirling Highway, Crawley, Perth, WA 6009, Australia; Department of Cardiology, Royal Perth Hospital, Victoria Square, Perth, WA 6000, Australia

**Keywords:** Calcific aortic valve stenosis, Aortic valve calcification, Aortic valve replacement, Vitamin supplementation, Randomized controlled trial, Vitamin K1

## Abstract

**Aims:**

Calcific aortic stenosis (CAS) is the most common heart valvulopathy in high-income countries. There is no known treatment for CAS other than replacement of the valve in severe, symptomatic disease. Observational studies and a small openlabel randomized trial have reported that vitamin K1 supplementation may reduce the progression rate of calcification and obstruction in CAS.

**Methods and results:**

PASSPORT(ACTRN12622000447752) will be a prospective, randomized, double-blind, placebo-controlled clinical trial investigating if nutritional supplementation with 10 mg of vitamin K1 can reduce the rate of valvular calcification and haemodynamic progression in CAS. Patients identified to have mild or moderate CAS based on standard echocardiographic criteria will be randomized 1:1 to vitamin K1 10 mg per day or matched placebo, and followed-up for a mean period of 16 months, ranging from 12 to 21 months. The primary endpoint will be the difference in aortic valve calcification volume, measured by computed tomography aortic valve calcium score, from baseline to follow-up, and secondary endpoints will include the change in echocardiographic progression of CAS, including peak flow velocity, mean pressure gradient, and aortic valve area. The trial is registered with the Australian New Zealand. Clinical Trials Registry (ACTRN12622000447752). The trial has met its recruitment target of 108 participants.

**Conclusion:**

PASSPORT will be prospective, randomized, double-blind clinical trial powered to demonstrate if oral supplementation with vitamin K1 reduces the progression of valvular calcification and echocardiographic severity of disease in patients with non-severe CAS. The trial results will have implications for the management of CAS, for which there is currently no medical treatment.

## Graphical Abstract

**Graphical Abstract:**
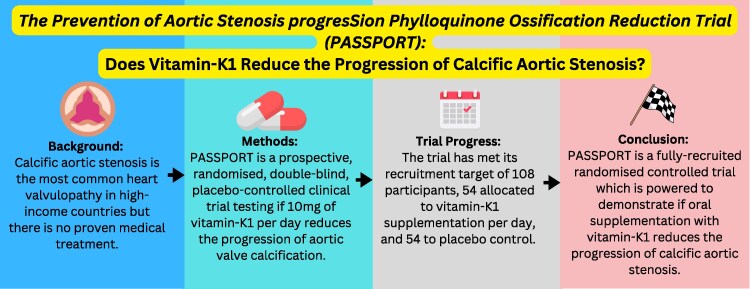

## Background and rationale

Calcific aortic valve stenosis (CAS) is the leading cause of valvular heart disease-related mortality and the third most frequent cardiovascular disease, after coronary artery disease and systemic hypertension, in high-income countries.^1^ For patients with severe and symptomatic disease, the risk of death within 2 years exceeds 50% in those who do not receive valve replacement,^2^ and the mortality rate of patients with CAS regardless of severity is higher than the general population.^3^ The prevalence of CAS increases with age, affecting 1% of patients aged 60 to 69 years and 10% of those aged 80 to 89 years.^4^ Whilst the burden of this deadly disease is increasing in tandem with the ageing population, the only effective treatment is replacement of the stenotic valve, either surgically or using a transcatheter approach, which both confer significant risk of peri- and post-procedural complications, and have a high cost.^5^ There is a clear need to develop a pharmacotherapy to prevent or reduce the progression of this valvulopathy.

The build-up of calcium on the aortic valve is the main driver of CAS. Indeed, the degree of aortic valve calcification (AVC) in patients with mild or moderate CAS is a key predictor of disease progression.^6,7^ The development of AVC is a complex, metabolically active, cell-mediated process, regulated by multiple genetic and environmental factors.^8,9^ Shear stress and other risk factors cause valvular endothelial injury and subsequent extravasation of lipids into the interstitium. These lipids are then oxidized in a process which promotes inflammation, differentiation of valvular interstitial cells into myofibroblasts and osteoblast-like cells, and calcification.^10^ The detection of these early microcalcifications using positron emission tomography scans predicts the later development of macroscopic calcifications, detectable on computed tomography (CT) scans, within 2 years.^11,12^ The progressive calcification of valvular tissue may ultimately cause transvalvular flow obstruction. Multiple trials using various pharmacotherapies to target this calcification pathway and its upstream promoters using agents, such as statins,^13–16^ renin–angiotensin–aldosterone system blockers,^17–19^ and anti-osteoporosis treatments (bisphosphonates, RANKL inhibition), have failed to show a reduction in CAS progression.^20^

Vitamin K prevents vascular calcification through the activation of matrix gamma-carboxyglutamic acid (GLA) protein and GLA-rich proteins, both of which are present in the leaflets of the aortic valve.^21,22^ It is thought that the vitamin K-dependent carboxylation of the matrix GLA protein stabilizes extracellular vesicles produced by valvular interstitial cells and thereby reduces calcification.^21,23^ Even in well-nourished people, supplementation with vitamin K increases the levels of carboxylated matrix GLA protein^24^ which should in theory increase its anti-calcific properties. Ueland and colleagues demonstrated that levels of serum undercarboxylated matrix GLA protein were positively associated with mortality in patients with severe, symptomatic CAS,^25^ and our group showed that low dietary vitamin K is associated with increased risk of cardiovascular disease related hospitalizations.^26^ Indeed, antagonism of vitamin K by warfarin accelerates the progression of CAS through a reduction in the activity of these anti-calcific, vitamin K-dependent proteins.^27–29^

Vitamin K refers to a group of fat-soluble vitamins which share a common 2-methyl-1,4-napthoquinone structure but differ in the length and saturation of their prenylated side chain. The two most important isoforms are vitamin K1 (phylloquinone) and vitamin-K2 (menaquinone). A recent randomized controlled trial conducted by Diederichsen and colleagues showed that supplementation with vitamin K2 did not influence the progression of AVC.^30^ In contrast, Brandenburg and colleagues demonstrated in a prospective, open-label, randomized, controlled trial that vitamin K1 supplementation, 2 mg/day, did reduce the rate of AVC progression, as measured by CT AVC quantification, when compared to placebo.^31^ More recently, we reported that a high intake of vitamin K1-rich foods was associated with reduced risk of incident CAS in a Danish population of >55 000 patients.^32^ These data suggest that supplementation with vitamin K1 may prevent the development or reduce the progression of CAS.

The primary aim of the Prevention of Aortic Stenosis Progression Phylloquinone Ossification Reduction Trial (PASSPORT) is to determine if supplementation with oral vitamin K1 can prevent the progression of AVC in patients with mild or moderate CAS. Additionally, we will determine if supplemental vitamin K1 can attenuate the echocardiographic progression of CAS, as measured by indices of disease severity, including peak flow velocity, mean pressure gradient, and aortic valve area.

## Methods and analysis

### Study design, inclusion and exclusion criteria

**Figure 1:**
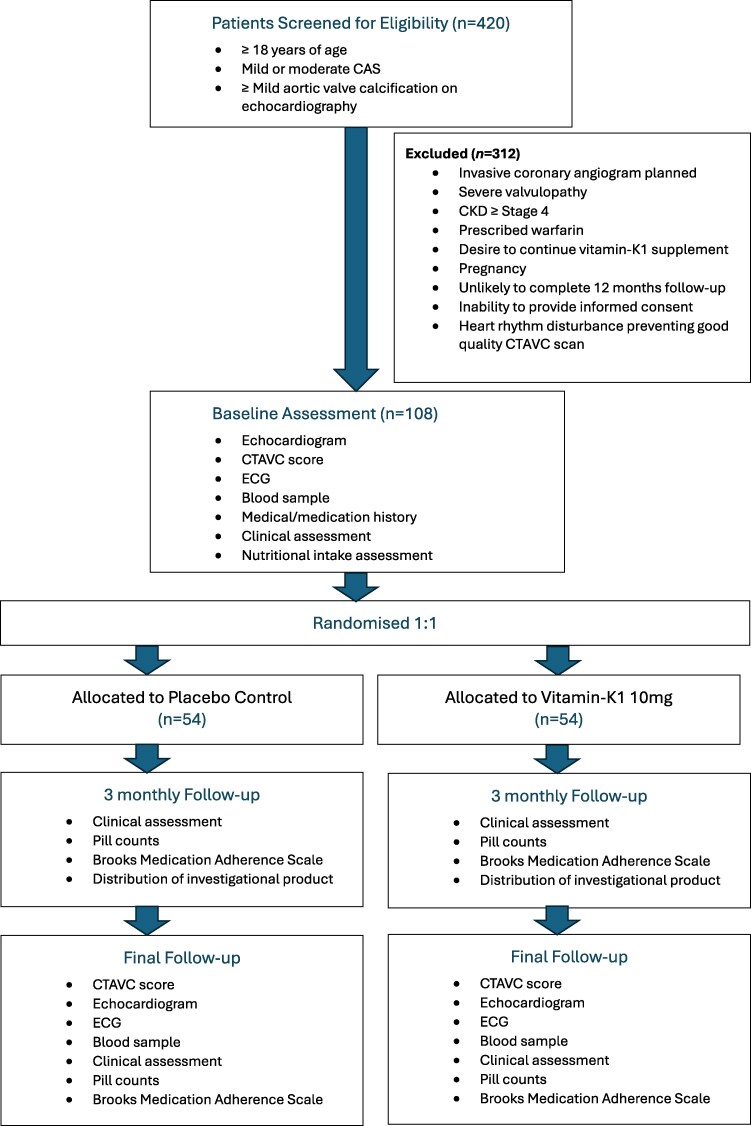
Flow diagram of trial participant selection. Abbreviations: *CAS*, calcific aortic valve stenosis; *CKD*, chronic kidney disease; *CTAVC*, computed tomography aortic valve calcification; *ECG*, electrocardiogram.

PASSPORT will be a prospective, investigator-initiated, single-centre, double-blind, randomized, controlled trial, conducted in Royal Perth Hospital, Australia. A total of 108 adult participants with mild or moderate aortic stenosis will be randomized 1:1 to either vitamin K1 or a matched placebo for an average of 16 months (*Figure 1*).

Patients will be included if they are ≥ 18 years, have mild or moderate CAS, defined echocardiographically by peak flow velocity ≥ 2.0 m/s and ≤ 4.0 m/s (mild CAS is defined as peak flow velocity ≥ 2.0 m/s and ≤ 3.0 m/s; moderate CAS is defined as peak flow velocity ≥ 3.1 m/s and ≤ 4.0 m/s),^33^ have an aortic valve area >1.0m^2^, ^33^ and have at least mild AVC, defined by Rosenhek category ≥ 2.^34^

Patients will be excluded from the trial if they are planned to have an invasive coronary angiogram that may lead to revascularization, have a severe valvulopathy of any heart valve, stage 4 or stage 5 chronic kidney disease (estimated glomerular filtration rate <30 mL/min), are prescribed a vitamin K1 antagonist, wish to continue taking vitamin K1 supplementation, are pregnant, are unlikely to be able to complete 12 months of follow-up, are unable to give informed consent, have a heart rhythm disturbance preventing a good quality CT-AVC scan (*Table 1*).

**Table 1:** Inclusion and exclusion criteria

**Inclusion criteria**
Aged ≥ 18 years Mild or moderate calcific aortic valve stenosis (defined echocardiographically by peak flow velocity ≥2.0 m/s and ≤ 4.0 m/s)^33^ Aortic valve area >1.0m^2^ ^33^ At least AVC on echocardiogram (Rosenhek category ≥2)^34^
**Exclusion Criteria**
Planned coronary angiogram Severe valvulopathy of any heart valve Stage 4 or stage 5 chronic kidney disease (estimated glomerular filtration rate <30 mL/min) Prescribed vitamin K1 antagonist A desire to continue taking a vitamin K1 supplement Pregnancy Unlikely to complete 12 months of follow-up Inability to provide informed consent Heart rhythm disturbance preventing a good quality CT-AVC quantification scan

AVC, aortic valve calcification; CT, computed tomography.

### Source of participants

Though recruitment and other trial related activities will be undertaken at single centre (Royal Perth Hospital), potential participants will be identified by clinicians in hospitals and private cardiology clinics within Western Australia. Participants will be initially screened using a transthoracic echocardiogram, performed for routine clinical purposes. If the most recent echocardiogram is more than three months old at the time of recruitment to the trial, the scan will be repeated to ensure that the documented severity of CAS at the time of enrolment is accurate. Following a diagnosis of mild or moderate CAS on transthoracic echocardiography, potential participants will be screened for eligibility using the pre-specified inclusion/exclusion criteria. Patients deemed ineligible for the study will be screen-failed, and their details will be stored on a de-identified screening log in the investigator site file.

### Blinding and randomization

Eligible patients who provide informed consent to participate in the study will be randomized to receive vitamin K1 or matching placebo. The randomization schedule will be generated using a 1:1 allocation ratio and random block sizes of two or four to either active treatment or placebo. The randomization will be stratified by CAS severity (either mild or moderate) as determined by the peak flow velocity measured on transthoracic echocardiography, to ensure that the treatment and placebo arm have an equal distribution of participants with mild and moderate CAS. The randomization allocation for each participant will be performed by the Royal Perth Hospital clinical trials pharmacy. Participants, referring physicians, trial researchers, and study personnel assessing outcomes will be blinded to treatment allocation.

### Intervention

A recent study demonstrated that participants with higher vitamin K1 intakes had a significant reduction in risk for CAS with subsequent aortic valve replacement, heart failure, or cardiovascular death.^32^ In that study, vitamin K1 intakes (median 191.6 µg/day) among participants were much higher than the currently recommended dosages.^35,36^ A randomized, controlled trial previously showed that a dose of 2 mg of vitamin K1 reduced the rate of progression of AVC in a cohort of patients with CAS and aortic sclerosis over a period of 12 months.^31^ In the PASSPORT trial, patients with aortic sclerosis will be excluded and only those with at least mild CAS will be included. The dose of vitamin K1 supplementation will be 10 mg because it is likely that participants in the PASSPORT trial will have advanced valvular calcification, necessitating a higher therapeutic dose of vitamin K1 to retard disease progression. Furthermore, 10 mg of vitamin K1 supplementation is safe to use in patients and a previous trial documented no adverse effects.^37^ Participants will spend a mean time period of 16 months on the PASSPORT trial.

The vitamin K1 10 mg capsules and matching placebo capsules (micro-cellulose) will be sourced from Nutrition Care Pharmaceuticals Ltd (Melbourne, Victoria, Australia). The investigational product will be packaged into bottles of 100 capsules by Nutrition Care and will be stored and dispensed by the clinical trials pharmacy of Royal Perth Hospital.

### Trial duration/schedule

The planned schedule of events for the trial is outlined in *Table 2*. After providing consent for the study, participants will attend a baseline visit. At the baseline visit, information regarding demographics, comorbidities, medications, and supplements will be collected. Dietary nutritional intake will be assessed using the Cancer Council Victoria Dietary Questionnaire for Epidemiological studies (DQES V3.2).^38^ The baseline clinical assessment will include a recording of height, weight, and blood pressure. To improve accuracy, blood pressure measurements will be taken using an automated sphygmomanometer and in the sitting position.

**Table 2:** Schedule of events for baseline, 3-monthly follow-up, and final visits

Study event	Baseline visit	3-monthly follow-up	Final visit
Echocardiogram	X		X
CTAVC scan	X		X
Demographics	X		
Comorbidities	X		
Medications	X		
Supplements	X		
Clinical examination	X		X
Dietary intake (DQES v3.2)	X		
Blood sample	X		X
IP distributed	X	X	
IP collected		X	X
Pill count		X	X
BMAS		X	X
Randomization	X		

BMAS, Brooks Medication Adherence Scale; CTAVC, computed tomography aortic valve calcification; DQES v3.2, Dietary Questionnaire for Epidemiological Studies Version 3.2; IP, investigational product.

Baseline investigations will include a 12-lead electrocardiogram (ECG); blood tests, including serum creatinine, corrected calcium, phosphate, and cholesterol profile, and the ratio of serum undercarboxylated osteocalcin to total serum osteocalcin (ucOC/tOC), an established method of measuring vitamin K status;^39,40^ a standardized transthoracic echocardiogram according to current guidelines;^41^ and a standardized low-dose, ECG-triggered, non-contrast CT-AVC score scan, where image quality will be optimized by achieving a target heart rate <60 beats per minute, as per the local clinical protocols.^42^ Acquisition parameters for the CT-AVC will include a minimum 64-slice scanner, 120–140KV with tube current modulated according to body weight. Image reconstruction will be performed in the diastolic phase (60–80% of the RR-interval) at both 3 and 1 mm slice thickness. Radiation dose will be minimized by using prospective tube current modulation and other techniques.

Once enrolled in the trial, participants will be randomized to either vitamin K1 or placebo and given a 3-month supply of the investigational product. Participants will be given a contact number and email address for the trial coordinator and requested to report any interruptions to treatment. They will also be also advised to avoid taking vitamin K1 supplements and to inform the trial coordinators if they are prescribed warfarin. The community doctor and cardiologist for each participant will be informed in writing about recruitment to the trial and provided with contact details for the trial coordinator.

### Follow-up and trial termination

Participants will be required to present in person every 3 months for clinical review. Changes to medications and important clinical updates, such as diagnosis of new medical conditions, admission to hospital, and any potential adverse events related to the investigational product will be recorded. At each follow-up visit, participants will be supplied with investigational product for 3 months. Follow-up of participants will continue for a minimum of 12 months (mean 16 months, maximum 21 months, *Figure 1*).

At the final follow-up visit, all participants will have a repeat clinical assessment and investigations which were performed at baseline (*Table 2*).

### Adherence and withdrawal from trial

To assess adherence to the investigational product, participants will complete the Brooks Medication Adherence Scale, and pill counts will be performed at each follow-up visit. The Brooks Medication Adherence Scale will be used due to its concise, 4-question, yes/no format which will provide information on both unintentional and intentional non-adherence to the investigational product. Additionally, the change in ucOC/tOC from the baseline and final visits will be compared which will provide information on adherence to the vitamin K1 for participants in the treatment arm.

Participants may leave the study by withdrawing their consent to participate at any time and for any reason. Any data collected until the date of withdrawal will be retained and used in the final analysis.

### Study outcomes

The primary outcome of the trial is progression of AVC volume score measured by non-contrast cardiac CT.^43^ For each participant, the annualized progression of AVC volume will be calculated as (follow-up AVC minus baseline AVC) ÷ time between scans in decimal years. Secondary outcomes will include the annualized progression in AVC density and AVC Agatston;^44^ changes in standard transthoracic echocardiographic indices of CAS severity, including peak transvalvular velocity, mean pressure gradient, aortic valve area, and aortic valve dimensionless index and the change in left ventricular mass, between baseline and study end. Other outcomes will include an analysis of the safety profile of high-dose vitamin K1 intake.

All study outcomes and clinical events will be assessed by an independent committee, blinded to treatment allocation.

### Statistical considerations

The primary intention-to-treat analysis will evaluate the within-group change in CT-derived AVC volume scores between baseline and study end (mean 16 months, minimum 12 months, maximum 21 months), using a repeated-measures general linear model. The model will include fixed effects for treatment group (vitamin K1 10 mg/day vs. placebo), duration of follow-up (baseline to final visit), and their interaction (treatment × duration of follow-up), with subject as a random effect. Baseline covariates (e.g. age, sex, baseline AVC score) will be included to adjust for potential confounders. The *treatment × time* interaction will estimate and test the between-group difference in mean AVC change, assessing whether the active treatment significantly attenuates AVC progression compared to placebo. Model assumptions (normality, homogeneity of variances) will be verified using diagnostic plots and tests, with transformations applied if needed.

A prior open-label study, using CT-derived AVC volume quantification, reported a 103μL (57%) lower AVC progression in 38 subjects who received vitamin K1 (2 mg per day) for 12 months when compared with 34 control subjects.^31^ Based on this, the PASSPORT trial, which will have 54 subjects per study arm (total 108), will provide 90% power to detect a 57% difference in AVC progression (181 μL in controls vs. 78 μL for active treatment) between the groups, assuming a common standard deviation of 165 μL. This sample size will also provide 80% power to detect a 49% difference and 70% power to detect a 40% difference, at a significance level of 5%. We anticipate a low dropout rate of 5–10% based on previous experience with similar cardiovascular trials,^37^ supported by robust follow-up procedures and regular participant contact. However, in the event of a loss to follow up of up to 25% (from 54 to 41 subjects per arm), the trial would retain 80% power to detect the primary effect (57% difference in AVC progression).

### Adjudication of outcomes

Adjudication of all outcomes will be performed by investigators who will be blinded to the treatment allocation. The Image analysis of the CT-derived AVC volume scores for the study will be performed centrally by two independent imaging specialists, also blinded to the treatment allocation. AVC volume scores^43^ will be calculated using vendor independent software (OsiriX, Version 5.8.5).

### Safety measures

The risks associated with participation in this research project are expected to be minimal.^37^ However, all adverse events will be systematically documented and reported in accordance with the Good Clinical Practice guidelines.

Participants will be reviewed at 3-month intervals throughout the study period to monitor clinical status and assess for any emerging concerns. The trial team will be specifically vigilant for increased risk for developing venous thromboembolism and blood dyscrasias. Participants will receive a dedicated contact phone number and email address to reach the study coordinators directly for support of queries. They will be requested to notify the trial team promptly in the event of hospital admission or any significant health event. The primary care physician for each participant will be informed of their enrollment in the trial and provided with contact details for the trial coordination team.

These measures are intended to ensure timely identification and appropriate management of any safety issue, which might arise during the trial.

## Study status

The protocol for the PASSPORT trial was approved by the human research ethics committees of Royal Perth Hospital and the East Metropolitan Health Service (Project Reference Number: RGS 0000005161) and is registered with the Australian New Zealand Clinical Trials Registry (ACTRN12622000447752). Recruitment for the PASSPORT trial began in July 2023 and was completed on 01 July 2024. By that date, 108 participants were randomized into the trial. The baseline characteristics of the participants randomized to PASSPORT are described in *Table 3*.

**Table 3:** Baseline characteristics of participants randomized into the PASSPORT trial

Characteristic	Randomized patients (*n* = 108)
Demographics	
Age (years), mean (SD)	73 (± 8.9)
Male, *n* (%)	73 (67.6%)
CAS severity (as per pVel on TTE)	
Mild, *n* (%)	74 (68.5%)
Moderate, *n* (%)	34 (31.5%)
Initial CTAVC score (Agatston units^a^), Median (IQR)	716 (413–1424)
Hypertension, *n* (%)	71 (65.7%)
Diabetes mellitus, *n* (%)	26 (24.0%)
Hypercholesterolemia	64 (59.3%)
BMI (kg/m2), mean (SD)	28.7 (± 4.8)
Current smoker, *n* (%)	4 (3.7%)
Medication use at baseline	
ACEi/ARB, *n* (%)	67 (62.0%)
Statin, *n* (%)	66 (61.1%)
Beta-blocker, *n* (%)	30 (27.8%)
NDP CCB, *n* (%)	5 (4.6%)

^a^Though Agatston units is provided in this table of baseline characteristics, the primary outcome of the PASSPORT trial is change in calcium volume. Change in Agatston units is a secondary outcome.

*ACEi*, Angiotensin-converting-enzyme inhibitor; *ARB*, angiotensin receptor blocker; *BMI*, body mass index; *CAS*, calcific aortic valve stenosis; *CKD*, chronic kidney disease; *CTAVC*, computed tomography aortic valve calcification; *NDP-CCB*, non-dihydropyridine calcium channer blocker; *pVel*, peak blood flow velocity through aortic valve; *SD*, standard deviation; *TTE*, transthoracic echocardiography.

As of 6 March 2025, four participants have dropped out of the trial, three required aortic valve replacement surgery, and one was diagnosed with stage 4 cancer. A total of 104 participants continue on the trial. No adverse effects related to the investigational product have been identified.

## Discussion

The PASSPORT Trial will provide information on the efficacy of oral vitamin K1 supplementation for the secondary prevention of CAS. A previous open-label randomized controlled trial which showed that vitamin K1 reduced AVC progression analysed data from 72 patients and included participants with aortic sclerosis.^31^ The PASSPORT trial aims to build on this knowledge by using a double-blind approach, recruiting a larger participant cohort (*n* = 108), increasing the duration of follow-up (mean participant duration of 16 months), and including only patients who have mild or moderate CAS.

A potential limitation of this trial is confounding by dietary vitamin K1 intake and baseline vitamin K1 status, which may vary between participants and might obscure the effect of vitamin K1 supplementation. To address this, baseline and final blood tests will measure the ucOC/tOC, a validated biomarker of vitamin K1 status. Each participant will also have a baseline dietary nutritional intake assessment, using the DQES V3.2 questionnaire,^38^ which has been developed and validated for assessing food and nutrient intakes in Australia.^45,46^ Although the DQES V3.2 does not directly quantify vitamin K1 intake, estimated intake will be derived from key foods using published nutrient databases. Baseline ucOC/tOC and estimated dietary vitamin K1 intake will be included as covariates in the repeated measures general linear model alongside other baseline covariates to adjust for potential confounding. Exploratory sensitivity analyses will assess treatment effect heterogeneity by baseline vitamin K1 status to evaluate if dietary or baseline status attenuates the effect of supplementation. Another potential limitation of the trial is the use of non-contrast CT. CT AVC quantification correlates well with CAS disease severity in most patients,^47^ but this modality does have some limitations. Approximately a quarter of individuals with severe CAS, particularly among females, younger patients, and those with a bicuspid valve, have lower levels of valvular calcification because in these cohorts the disease is predominantly driven by fibrotic change.^7,48^ However, CT demonstrates excellent scan-rescan reproducibility when measuring AVC volume, density, and Agatston units and is therefore a dependable modality to measure AVC progression, which is the primary outcome of this trial. Whilst women likely will have less calcification at baseline and progress at a slower rate than men, the randomization is likely to ensure equal distribution of sex across treatment groups.^49^

It is well established that vitamin K is crucial for the maintenance of effective coagulation and the minimum daily intakes recommended by the National Institute of Medicine (90–120 µg/day) and the European Food safety Authority (70 µg/day)^35,36^ were formulated based on the presumption that haemostasis is the only metabolic process in which vitamin K is involved. The importance of this cofactor in maintaining the integrity of the vasculature has only been recognized relatively recently, and the exact daily dosage needed to optimize this remains unknown.^50,51^ Multiple studies have suggested that vitamin K1 supplementation reduces calcification not only in the aortic valve but also in the peripheral and coronary arteries.^25,26,32,52^ It is possible that the currently recommended daily intake may need to be revised to a higher dose.

PASSPORT is the only trial currently registered to test the hypothesis that vitamin K1 prevents calcification of the aortic valve.^32^ If vitamin K1 supplementation proves effective, it would be the first proven pharmacotherapy for the treatment of CAS and would dramatically alter the global therapeutic landscape for this deadly condition, where a high-risk, high-cost valve replacement is currently the only known definitive treatment. It is likely that oral supplementation with vitamin K1 would be incorporated into clinical practice and guidelines as a low-cost, safe, and effective treatment for non-severe CAS. It stands to reason that subgroups of patients who manifest a predominantly calcific phenotype of CAS, older and male patients,^7^ might benefit most from the anti-calcific properties of vitamin K1. The randomization was also stratified by severity of CAS, which would enable an exploratory analysis assessing if vitamin K1 is more effective in mild rather than moderate CAS. More broadly, if the PASSPORT trial has a positive result, it would enhance our understanding of the physiological role that vitamin K1 plays in optimizing vascular health and provide a rationale for further studies to examine any role in reducing the progression of calcification in other locations. If the trial is negative, it will provide an important answer in our quest to find a medical treatment for CAS and will guide researchers towards investigating other potential therapeutic targets for this disease.

## Lead author biography


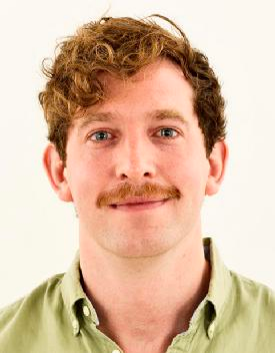


Dr William A. Courtney is a Doctorate of Philosophy candidate at the University of Western Australia. He has been awarded multiple scholarships for his research from the Australian government, including the Research Training Program Domestic Fees Offset Scholarship, a Future Health Research and Innovation Fund Clinician Researcher Training Scholarship, and the Athelstan Saw Top Up Scholarship. His research focuses on novel therapeutic targets in the treatment of aortic stenosis. Dr Courtney was identified as an Outstanding Emerging Cardiovascular Researcher by the Cardiac Society of Australia and New Zealand in 2023 and was a Cardiac Imaging Prize Finalist.

## Data Availability

The baseline characteristics table presented in this manuscript summarizes data from recruited participants in the PASSPORT trial. Individual participant data and additional datasets generated for the PASSPORT trial will be made available upon reasonable request to the corresponding author following completion of the study, subject to ethical approval and applicable data protection regulations. All data requests should specify the purpose of the data request and will be reviewed by the trial steering committee.
